# Responses of Different Temperature-Acclimated Diatom Species, Smaller *Thalassiosira pseudonana* and Larger *Thalassiosira rotula*, to Increased Ambient Temperature

**DOI:** 10.3390/microorganisms13071652

**Published:** 2025-07-12

**Authors:** Wei Zhao, Jihua Liu, Hui Song, Bokun Chen, Hongli Ji, Xue Yang, Gang Li

**Affiliations:** 1 Institute of Marine Science and Technology, Shandong University, Qingdao 266237, China; zhaowei417@hotmail.com (W.Z.); liujihua1982@sdu.edu.cn (J.L.); chenbokunimst@163.com (B.C.); jihongli2001@163.com (H.J.); 202416943@mail.sdu.edu.cn (X.Y.); 2 Joint Laboratory for Ocean Research and Education of Dalhousie University, Shandong University and Xiamen University, Qingdao 266237, China; 3 Qingdao Key Laboratory of Ocean Carbon Sequestration and Negative Emission Technology, Shandong University, Qingdao 266237, China; 4 Daya Bay Marine Biology Research Station, South China Sea Institute of Oceanology, Chinese Academy of Sciences, Shenzhen 518121, China

**Keywords:** temperature rise, ambient temperature, cell size, diatoms, *Thalassiosira*

## Abstract

The acute rise in temperature due to marine heatwaves has a strong impact on marine phytoplankton. To determine whether these effects depend on ambient temperature and cell size, we acclimated two diatom species, smaller *Thalassiosira pseudonana* (Hasle and Heimdal, 1970) and larger *Thalassiosira rotula* (Meunier, 1910), at low (LAT), medium (MAT) and high ambient temperatures (HAT) and examined their physiochemical and transcriptional responses to temperature rise (AT + 6 °C). The specific growth rate (µ) of smaller cells was increased by 32% due to temperature rise at LAT, but decreased by 13% at HAT, with the stimulatory and inhibitory extent being ~50% less than that of larger cells. At LAT, chlorophyll *a* (Chl *a*), carotenoid (Car) and carbon (POC) contents were increased in smaller cells due to temperature rise, but were decreased in larger cells; at HAT, Chl *a* and Car were increased in both smaller and larger cells and POC was increased in only smaller cells. At LAT, temperature rise led to a disproportionate increase in photosynthesis and dark respiration, resulting in an increase in carbon utilization efficiency (CUE) in smaller cells and a decrease in CUE in larger cells; at HAT, there was a decrease in CUE in both the smaller and larger cells, but to a lesser extent in the former than in the latter. Our results also show that smaller cells cope with the acute temperature rise mainly by strengthening their enzyme activity (e.g., the antioxidant system) and conservatively regulating their metabolism, while larger cells mainly regulate their photosynthetic and central carbon metabolism. Moreover, larger cells can outperform their smaller counterparts when the temperature rise occurs at lower ambient temperature.

## 1. Introduction

The world’s oceans have absorbed more than 90% of the total energy increase on Earth since the Industrial Revolution [1], and accordingly, global sea surface temperature has risen by ~0.93 °C in the period 2013–2022 [2] and is projected to further rise by 3–5 °C by the end of this century, as reported by IPCC AR6 (SSP3-7.0 and SSP5-8.5). As the oceans warm, marine heatwaves, an extreme climate event characterized by periods of at least 5 days in which the seawater temperature exceeds the 90th quantile of a baseline temperature time series [3], will become more dramatic, with temperatures rises of as much as 8–9 °C within a short period [4]. Marine heatwaves have been observed in almost all major ocean basin areas in the last decade [5], not only in low- and mid-latitude regions, e.g., the Northeast Pacific [6], the German Bight [3], the Indian Ocean [7] and the China Sea [8], but also in high-latitude regions, e.g., the Arctic Ocean [9]. The rise in temperature has a significant impact on marine ecosystems, as it damages vulnerable species by altering their physiological processes and thus causes a loss of marine biodiversity [2]. As one of the most important producers, marine phytoplankton live mainly in surface oceans to harvest solar energy for photosynthesis and are therefore inevitably affected by the rise in temperature, which in turn affects the provision of food for consumers higher up the chain and the support of marine ecosystems [10].

It is known that a rise in temperature can significantly affect the growth [11], cell composition [12] and even cell volume of marine phytoplankton [13] by altering their ability to absorb nutrients [14] and their physiological metabolism [15,16]. At low ambient temperature (LAT), cellular enzyme activity and membrane fluidity are restricted, limiting physiological processes such as photosynthesis, respiration, nutrient uptake and ultimately growth [17]. However, when the temperature exceeds an optimal range, phytoplankton often accumulate more reactive oxygen species (ROS) within their cells, which damages the cellular macromolecules and photosynthetic apparatus [18] by inactivating enzymes, e.g., Rubisco, leading to excess accumulation of excitation energy [17]. Therefore, a rise in temperature usually increases the primary productivity of phytoplankton in the cold Southern Ocean [11], as well as the biomass of *Pseudo-nitzschia, Phaeodactylum Tricornutum* and *Chlamydomonas reinhardtii* at LAT by up-regulating carbon assimilation, non-photochemical quenching and nitrogen assimilation [11,16,19]. The rise in temperature also enhances cellular organic carbon and nitrogen production of *Skeletonema dohrnii* through rapid evolution of genes associated with energy production and lipid metabolism [20]. However, at high ambient temperature (HAT), a further rise in temperature has adverse effects on the metabolic rate and net primary production of phytoplankton assemblages in the North Indian Ocean, Java Sea and southern South China Sea [21,22]. As marine heatwaves can occur not only in summer and at low latitudes [7,8] but also in other seasons and at high latitudes [5,9,23], it is thus essential to consider the effects of temperature rise on phytoplankton under different ambient temperatures and its regulatory mechanism.

It has been observed that the rise in temperature has led to the miniaturization of the phytoplankton community [13,24]. However, the physiological mechanisms underlying this phenomenon are still unclear, despite the importance of understanding the effects of rising temperatures on phytoplankton. As a representative group of marine phytoplankton, diatoms are widely distributed in coastal waters from tropical to cold oceans and contribute to about 40% of marine primary productivity [25,26,27]. The cell size of diatoms exhibits a wide range, with a range in effective diameter from 2 to 2000 μm [28] and a difference in cell volume of more than nine orders of magnitude [24,28,29], which can lead to differences in light absorption capacity [30], nutrient uptake kinetics [31], metabolic rate [32], etc. The acclamation of smaller and larger phytoplankton cells to the temperature rise can therefore be achieved via different strategies. Smaller diatoms, such as *Pseudo-nitzschia* and *Thalassiosira pseudonana*, with a higher surface area-to-volume ratio often actively respond to the temperature rise by greatly increasing cellular pigment and biogenic silica content as well as Rubisco activity [11,29,33]. Larger diatoms such as *Thalassiosira punctigera* with a higher deactivation energy are more susceptible to temperature rise, which is reflected in a significant reduction in growth and cell volume [29,34]. However, most previous studies focused on the effects of temperature rise at a given temperature on smaller or larger diatoms, so it is still unclear whether the cell size-dependent dynamics of responses to temperature rise are related to ambient temperature.

Our previous results showed that the responses of the natural phytoplankton community to temperature rise are very different in different seasons, as evidenced by the larger cells benefiting more from the temperature rise in winter and the smaller cells tolerating it more in summer [35]. To uncover the mechanism underlying this phenomenon, we acclimated two representative diatom species, the smaller *Thalassiosira pseudonana* (Hasle and Heimdal, 1970) and the larger *Thalassiosira rotula* (Meunier, 1910), at three ambient temperatures (ATs, i.e., 10 °C, 18 °C and 26 °C), shifted them to a rising temperature (AT + 6 °C) and examined their changes in physiological performance and transcriptome. This work contributes to gaining a better understanding of the different responses and adaptation strategies employed by marine phytoplankton with different cell sizes in response to a temperature rise at different ambient temperatures.

## 2. Materials and Methods

### 2.1. Culture Protocol

**Figure 1:**
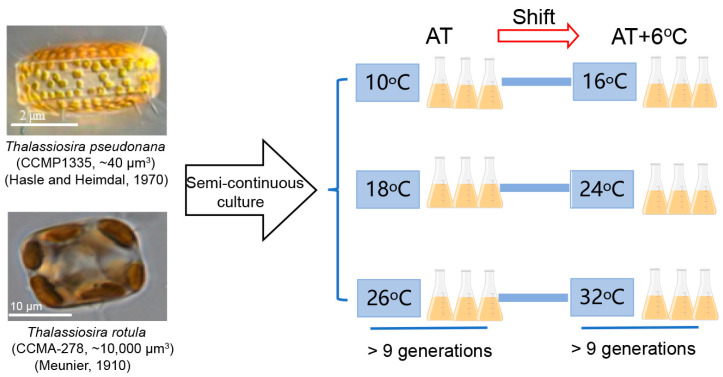
The flow scheme of the experiment.

The marine centric diatoms *Thalassiosira pseudonana* (CCMP 1335) from the Provasoli-Guillard National Center of Marine Phytoplankton (NCMP) and *Thalassiosira rotula* (CCMA-278) from the Center for Collections of Marine Algae (CCMA) were initially kept at 18 °C with semi-continuous culture in a growth chamber (ZQZY-CGF8, Shanghai Zhichu, Shanghai, China) in 500 mL conical flasks filled with sterilized enriched artificial seawater (ESAW) [36]. These two diatom species are globally distributed in temperate marine areas [37] with a cell volume of ~40 μm^3^ for *T. pseudonana* [38] and ~10,000 μm^3^ for *T. rotula* [39]. Their cellular dimensions were ~3 μm × ~2 μm (diameter × height) and ~20 μm × ~10 μm, respectively (Figure 1). The light in the chamber was set to an optimal level of 150 μmol photons m^−2^ s^−1^ [40], provided by a panel of white LED lights, and was automatically turned on at 08:00 and off at 20:00 to create a 12:12 light/dark cycle. During the experimentation, the cultures were acclimated at three ATs with semi-continuous culture, i.e., 10, 18 and 26 °C, followed by shifting to AT + 6 °C. The rates of photosynthesis and dark respiration were measured until they had acclimated to the growth condition after division over 9 generations at the end of the initial period of acclimation and under AT + 6 °C conditions [38,40]. Then, physiological measurements were performed. Meanwhile, all flasks were randomly positioned to eliminate possible biases due to light variation in the chamber. Three independent replicate cultures were used for each treatment.

### 2.2. Growth Rate

To assess the growth of *T. pseudonana* and *T. rotula*, the optical density of the cultures was measured daily at 10:00 a.m. (2 h after the lights were switched on) at 680 nm (OD_680_) using an ultraviolet–visible spectrophotometer (UV-1800, Shimadzu, Kyoto, Japan) before and after dilution with fresh medium. During cultivation, OD_680_ was maintained at 0.085 ± 0.011 and 0.028 ± 0.004, with a Chl *a* concentration of 0.72 ± 0.10 μg mL^−1^ and 0.30 ± 0.05 μg mL^−1^, respectively. The specific growth rate (μ, d^−1^) was calculated as follows:

(1) $$\mu =\frac{\ln\left(N_{t}\right)-\;\ln(N_{0})}{t\;-\;t_{0}}$$

where N_t_ and N_0_ represent the value of OD_680_ at time t and t_0_, respectively.

After the cultures had grown through at least 9 generations, duplicate 2 mL cultures of *T. pseudonana* and *T. rotula* were taken from each flask after careful shaking and fixed with glutaraldehyde to a final concentration of 1% (*v*/*v*) for cell counting using an Accuri C6 flow cytometer (Becton-Dickinson, Franklin Lakes, NJ, USA). Based on the linear correlation between the signal intensity of the FSC channel and cell size [41], the observed changes in cell volume were standardized to per volume for cellular biochemical parameters. After this, aliquots of 50–80 mL cultures were harvested to measure cell composition, physiology and transcriptome, as described below.

### 2.3. Cell Composition

Pigment concentration was quantified with 50 mL cultures of *T. pseudonana* and *T. rotula* filtered onto PC filter with a 0.2 μm pore size (25 mm in diameter, Millipore, Burlington, MA, USA). The filters were then extracted overnight at 4 °C in the dark with 5 mL of 90% acetone solution (*v*/*v*) saturated with magnesium carbonate (MgCO_3_). After centrifugation at 4000× *g* for 10 min at 4 °C, the optical absorbance of the supernatant was measured in the wavelength range of 400–750 nm using an ultraviolet–visible spectrophotometer (UV-1800, Shimadzu, Kyoto, Japan). Chlorophyll *a* (Chl *a*) and carotenoid (Car) concentrations were calculated as follows [42]:

(2) $$\mathrm{Chl}\;a=11.47\;\times \;\left(A_{664}\;-\;A_{750}\right)\;-\;0.4\;\times \;(A_{630}\;-\;A_{750})$$

(3) $$\mathrm{Car}=2.11\;\times \;\left(A_{630}\;-\;A_{750}\right)\;-\;10.01\;\times \;(A_{645}\;-\;A_{750})\;+\;4.37\;\times \;(A_{470}\;-\;A_{750})$$

where A_470_, A_630_, A_645_, A_664_ and A_750_ indicate the absorbance at 470 nm, 630 nm, 645 nm, 664 nm and 750 nm, respectively.

To measure cellular carbon content, 50 mL of cultures was filtered onto pre-combusted (450 °C for 5 h) GF/F filters (25 mm, Whatman, Buckinghamshire, UK). The filters containing cells were then exposed to HCl fumes for 3 h and then vacuum freeze-dried (Biocool, Beijing, China) for 24 h to remove inorganic carbon. The particulate organic carbon content (POC) was measured using an innovative elemental analyzer (NC Technologies, Marseille, France) with methionine (NC Technologies, Marseille, France) as the standard. Additionally, triplicate 50 mL EASW was filtrated on the pre-combusted GF/F filters as blank.

For cellular malondialdehyde (MDA) content and superoxide dismutase (SOD) activity assays, 50 mL of cultures was filtered onto a PC filter. The filters with cells were immersed in 1 mL of pre-chilled extraction buffer (pH 8, 20 mM Tris, 1 mM EDTA, 10 mM MgCl_2_, 50 mM NaHCO_3_ and 5 mM β-mercaptoethanol) and oscillated for 20 min at 4 °C with grinding beads to lyse the cells with a vortex mixer. After centrifugation at 1000× *g* for 10 min at 4 °C, MDA and SOD in the supernatant was measured with an MDA assay kit (A003-1, Nanjing Jiancheng Biological Engineering Co., Nanjing, China) and SOD assay kit (A001-3, Nanjing Jiancheng Biological Engineering Co.), respectively, following the manufacturer’s protocol [43].

### 2.4. Physiological Parameters

The photosynthetic rate and dark respiration rate were measured with 15 mL of culture taken from each flask and placed in a 15 mL chamber of the photosynthetic instrument (YZQ-201A, Yizongqi Technology Co., Ltd., Beijing, China). After dark acclimation for 15 min at each growth temperature, the increase in dissolved O_2_ concentration in the chamber under light and the decrease under dark were monitored using the YZQ-201A instrument. The net photosynthetic O_2_ evolution rate and dark respiration rate (Rd) were calculated by normalizing the rate of oxygen increase and decrease to the cell volume, expressed as 10^−3^ fmol O_2_ μm^−3^ min^−1^, and the gross photosynthetic O_2_ evolution rate (Pg) was obtained by summing the net photosynthetic O_2_ evolution rate and Rd. Carbon use efficiency (CUE) was assessed using Pg and Rd to estimate the fraction of photosynthates that are allocated to growth according to Allison et al. [44] as follows:

(4) $$\mathrm{CUE}=1\;-\;\frac{\mathrm{Rd}}{\mathrm{Pg}}$$

### 2.5. Transcriptome Sequencing and Analysis

The effects of temperature rise were analyzed according to the transcriptome profiles of T. *pseudonana* and *T. rotula* grown at 6 temperatures. In brief, 80 mL of cultures from each flask was filtered onto a Whatman GF/F filter. Total RNA from the collected cells was then extracted using Trizol (Takara Bio, Kusatsu City, Japan), and the quantity and quality of the total RNA were assessed by using nanodrop 2000 (Thermo Fisher Scientific, Waltham, MA, USA) and Agilent 5300 (Agilent Tech., Santa Clara, CA, USA), respectively. Only high-quality RNA samples with an OD_260/280_ ratio of approximately 2, a concentration of ≥30 ng μL^−1^ and an RQN > 6.5 were used for sequencing library construction. RNA purification, reverse transcription, library construction and sequencing were performed at Majorbio Biotech (Shanghai, China). The RNA-seq transcriptome library was prepared according to Illumina^®^ Stranded mRNA Prep Ligation (San Diego, CA, USA) using 1 μg of total RNA.

The raw paired-end reads were trimmed and quality-controlled using fastp [45] with default parameters. Subsequently, the clean data from the samples were used for de novo assembly with Trinity [46]. The assembled transcripts were annotated based on the following databases: NCBI non-redundant protein sequences (Nr), a manually annotated and reviewed protein sequence database (Swiss-Prot) and the Kyoto Encyclopedia of Genes and Genomes database (KEGG) using Diamond to identify the proteins with the highest sequence similarity. To identify differentially expressed genes (DEGs) responding to the temperature rise, the expression level of each transcript was calculated using the transcripts per million reads method. RSEM (RNA-Seq by Expectation Maximization) was used to assess gene transcription levels [47]. Differential gene expression analysis of temperature rise was performed using the DEGseq2 package [48]. Functional enrichment analysis of DEGs was performed using Python scipy software (1.0.0) and plotted using “ggplot2”, “ggrepel” and “ggbreak” packages in R software (4.4.2).

### 2.6. Statistical Analysis

All data were expressed as mean values ± the standard deviation (SD). To determine the effects of temperature increase, one-way ANOVA and a post hoc Tukey test were performed to test the null hypothesis that there are no differences between the physiological parameters (growth rate, Chl *a*, Car and POC content, photosynthetic rate, dark respiration rate, CUE, SOD activity and MDA content) of each diatom at different temperatures in SPSS 22.0. An FDR < 0.05 and |log_2_(FC)| > 1 were set as the significant threshold of DEGs.

## 3. Results

**Figure 2:**
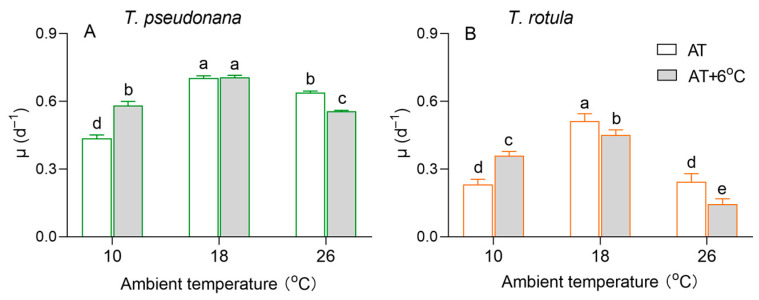
The specific growth rate (μ, d^−1^) of the smaller diatom *Thalassiosira pseudonana* (**A**) and the larger *Thalassiosira rotula* (**B**) at three ambient temperatures and the increased temperature. Values are the mean ± SD for triplicate independent cultures, and different letters above the bars indicate significant differences (*p* < 0.05, one-way ANOVA).

The growth of the smaller *T. pseudonana* and the larger *T. rotula* differed at different ambient temperatures, as did the effects of temperature rise (Figure 2). The specific growth rate (µ) of both smaller cells and larger cells showed a trend of first increasing and then decreasing with the increasing AT. The rise in temperature increased the µ of the smaller cells by 32% at LAT but decreased it by 13% at HAT (Figure 2A), and greater stimulatory (52%) and inhibitory effects (42%) occurred in larger cells (Figure 2B). In addition, the rise in temperature had no significant effect on the µ of smaller cells at MAT, although it reduced the µ of larger cells by 12%.

**Figure 3:**
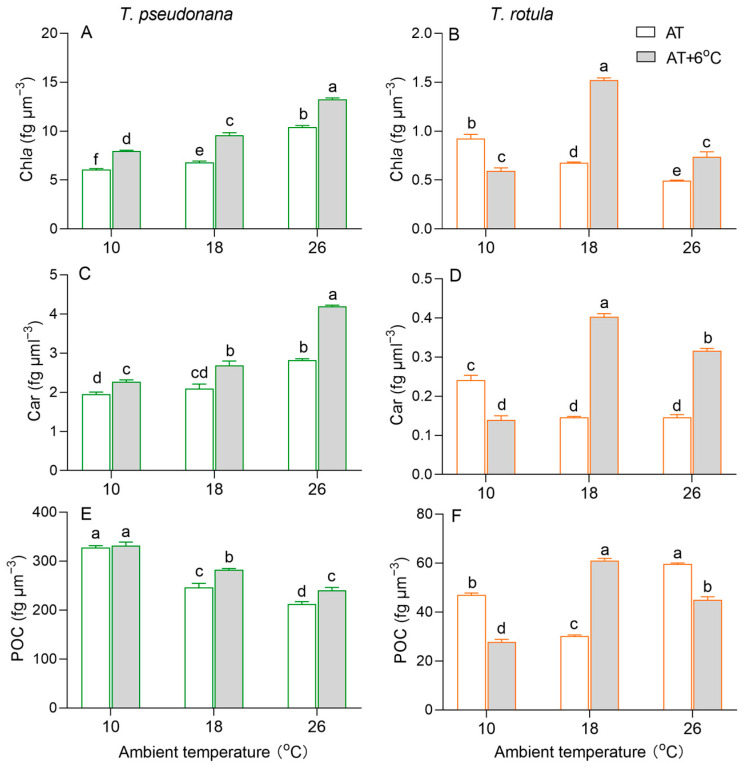
Cellular content (fg μm^−3^) of chlorophyll *a* ((**A**,**B**); Chl *a*), carotenoids ((**C**,**D**); Cars) and particulate organic carbon ((**E**,**F**); POC) of the smaller diatom *T. pseudonana* and the larger *T. rotula* at three ambient temperatures and the increased temperature. Values are the mean ± SD for triplicate independent cultures, and different letters above the bars indicate significant differences (*p* < 0.05, one-way ANOVA).

Chlorophyll *a* (Chl *a*) per cell volume increased in the smaller *T. pseudonana* with increasing AT, whereas it decreased in the larger *T. rotula* (Figure 3A,B). At LAT, the rise in temperature increased Chl *a* content in smaller cells by 32%, but decreased it in larger cells by 37%. At MAT and HAT, the rise in temperature increased Chl *a* in both smaller and larger cells. Similar to Chl *a*, carotenoids (Cars) increased in smaller cells with increasing AT, while they decreased in larger cells, as did the effects of the temperature rise (Figure 3C,D). Particulate organic carbon (POC) per cell volume decreased with increasing AT in smaller cells, whereas it firstly decreased and subsequently increased in larger cells (Figure 3E,F). At LAT, the rise in temperature had no significant effect on the POC content in smaller cells, but decreased it by 41% in larger cells. At MAT, the rise in temperature increased the POC content in both smaller and larger cells; at HAT, it increased the POC in smaller cells, while it decreased it in larger cells.

**Figure 4:**
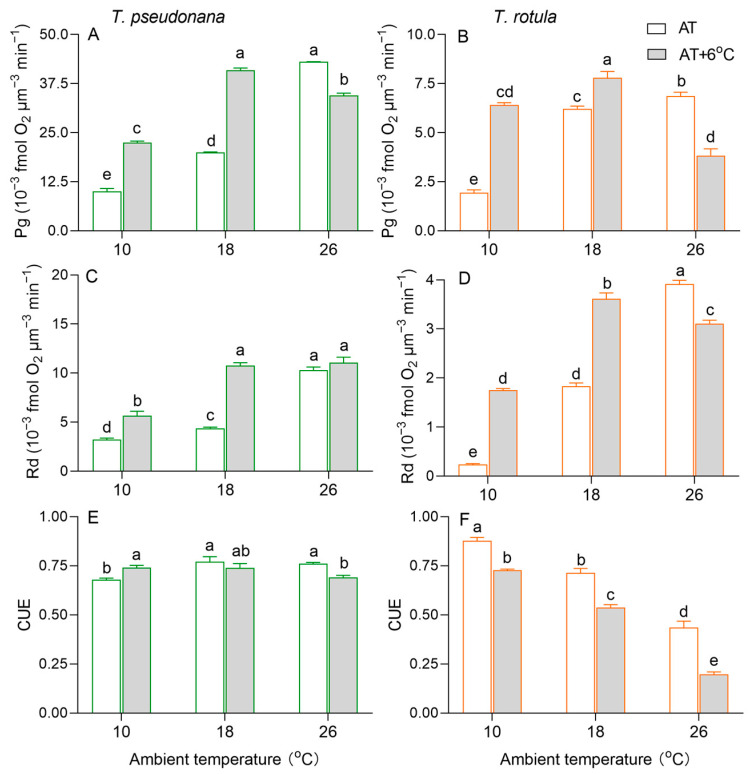
The rate (×10^−3^ fmol O_2_ μm^−3^ min^−1^) of gross photosynthesis ((**A**,**B**); Pg) and dark respiration ((**C**,**D**); Rd) and the carbon use efficiency ((**E**,**F**); CUE) of the smaller diatom *T. pseudonana* and the larger *T. rotula* at three ambient temperatures and the increased temperature. Values are the mean ± SD for triplicate cultures, and different letters above the bars indicate significant differences (*p* < 0.05, one-way ANOVA).

The gross photosynthetic O_2_ evolution rate (Pg) increased with increasing AT in both smaller and larger cells (Figure 4A,B). Due to the temperature rise, the Pg of the smaller cells was increased by 124% at LAT and by 105% at MAT, but it was decreased by 20% at HAT. Such stimulatory (i.e., 231% at LAT and 25% at MAT) and inhibitory effects (i.e., 43%) also occurred in larger cells. Similar to Pg, the dark respiration rate (Rd) increased with increasing AT in both smaller and larger cells, as did the effects of the temperature rise (Figure 4C,D). At HAT, the temperature rise had no significant effect on the Rd of smaller cells, but decreased it by 21% in larger cells. Moreover, we calculated the carbon use efficiency (CUE) to estimate the fraction of photosynthate allocated to growth. The CUE increased with increasing AT in smaller cells, while it decreased in larger cells (Figure 4E,F). At LAT, the rise in temperature increased the CUE in smaller cells but decreased it in larger cells. At MAT and HAT, the rise in temperature decreased the CUE in both smaller and larger cells, but was greater in the latter than in the former.

**Figure 5:**
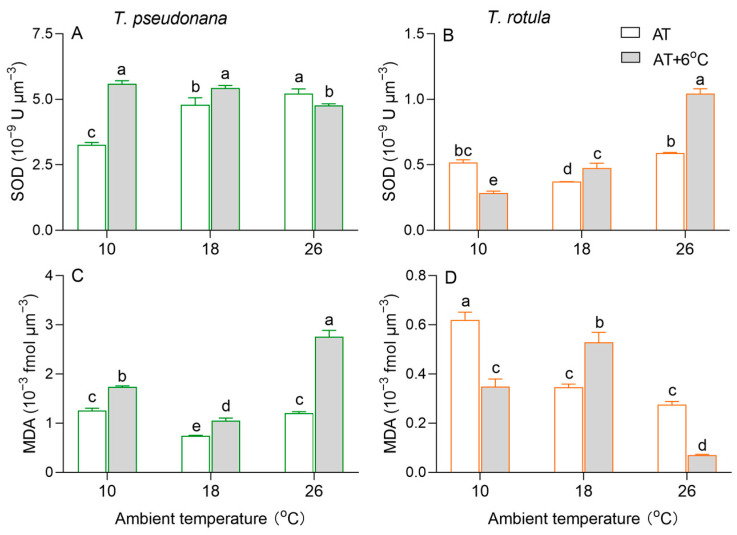
Cellular superoxide dismutase activity ((**A**,**B**); SOD, ×10^−9^ U μm^−3^) and malondialdehyde content ((**C**,**D**); MDA, ×10^−3^ fmol μm^−3^) of the smaller diatom *T. pseudonana* and the larger *T. rotula* at three ambient temperatures and the increased temperature. Values are the mean ± SD for triplicate independent cultures, and different letters above the bars indicate significant differences (*p* < 0.05, one-way ANOVA).

Cellular superoxide dismutase (SOD) activity per cell volume increased with increasing AT in smaller cells, whereas it firstly decreased and subsequently increased in larger cells (Figure 5A,B). The rise in temperature increased the SOD activity at LAT and decreased it at HAT in smaller cells, while an opposite trend was observed in larger cells. Malondialdehyde (MDA) per cell volume, a product of membrane lipid peroxidation, firstly decreased and subsequently increased in smaller cells, while it decreased with increasing AT in larger cells (Figure 5C,D). The rise in temperature increased MDA content by 38% at LAT and 126% at HAT in smaller cells, but oppositely decreased it by 47% and 89% in larger cells. At MAT, the rise in temperature increased MDA content in both smaller and larger cells.

**Figure 6:**
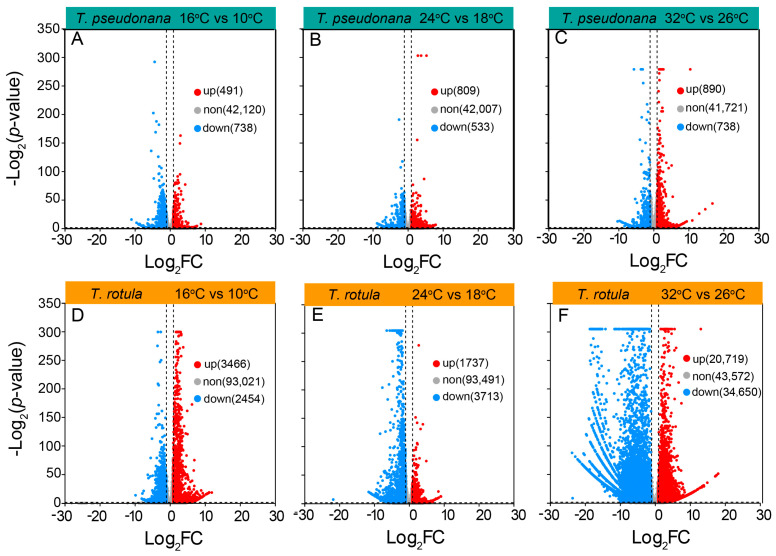
A volcano plot of differentially expressed genes (DEGs) in the smaller diatom *T. pseudonana* (**A**–**C**) and the larger *T. rotula* (**D**–**F**) responding to the increased temperature at three ambient temperatures.

To uncover the mechanisms underlying these different physiochemical responses to temperature, we analyzed the transcriptome profiles of the smaller *T. pseudonana* and the larger *T. rotula* grown at different ATs and elevated temperatures. In transcriptomic sequencing, a total of 120 Gb base data were obtained from 18 samples and annotated into 43,349 genes in smaller cells, and 126 Gb base data were annotated into 98,941 genes in larger cells. In smaller cells, the increased temperature-induced differentially expressed genes (DEGs) accounted for ~4% of the total genes, while the DEGs in larger cells accounted for ~60%. The number of down-regulated or up-regulated DEGs did not increase or increased only slightly in smaller cells when the AT increased (Figure 6A–C), whereas they increased strongly in larger cells (Figure 6D–F). In smaller cells, the rise in temperature led to a greater number of down-regulated DEGs at LAT, but fewer at MAT and HAT (Figure 6A–C); in larger cells however, there were fewer down-regulated DEGs at LAT, but more at MAT and HAT (Figure 6D–F).

**Figure 7:**
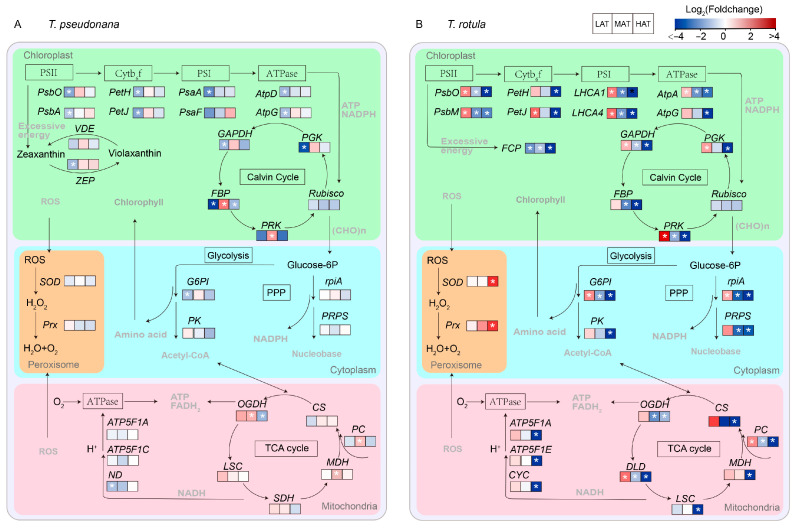
Transcriptomic schematic diagram of changes in carbon metabolic pathways of smaller *T. pseudonana* (**A**) and larger *T. rotula* (**B**) responding to increased temperature T three ambient temperatures. Significant differentially expressed genes (DEGs) (|log_2_FC| > 1, *p* < 0.05) are highlighted with white star. Details of DEPs are shown in Table S2.

The physiological functions of these DEGs were further analyzed based on KEGG annotation (Figure S1 and Table S1). The number of KEGG pathways decreased with increasing AT in both smaller (from 20 to 10) and larger cells (from 20 to 14) and the expression patterns of the KEGG enriched pathways clearly indicated that the transcriptional regulatory mechanisms of smaller cells and larger cells in response to temperature rise at different ATs are completely different (Figure S1). A transcriptomic schematic diagram of carbon metabolic pathways was created and analyzed, as shown in Figure 7. The expression of genes related to photosynthesis in smaller cells was generally transformed from down-regulation to conservation with increasing AT (Figure 7A and Figure S1), such as *PsbO*, *PsbA*, *PetH*, *PetJ, psaA psbO* and *AtpG* of photosynthesis (ko00195), *LHCA1* and *LHCA4* of photosynthesis-antenna (ko00196), and *FBP*, *PRK* and *PGK* of carbon fixation (ko00710), while these important pathways were generally transferred from up-regulation to down-regulation in larger cells (Figure 7B and Figure S1). On the other hand, genes relevant to central carbon metabolism appeared to be more sensitive to the temperature rise in larger cells than in smaller cells, such as *PK* and *G6PI* of glycolysis/gluconeogenesis (ko00564), *PC*, *OGDH* and *MDH* of the TCA cycle (ko00020) and *rpiA* and PRPS of the pentose phosphate pathway (ko00030). In addition, *SOD* and *Prx* of peroxisome (ko04146) exhibited no significant change in smaller cells, but up-regulation in larger cells, especially at HAT.

## 4. Discussion

The cell size-dependent responses of diatoms to environmental changes have been extensively studied. The rise in temperature promoted the growth of smaller diatoms to a greater degree compared to larger diatoms [11], regulated the cell pigments of smaller diatoms more than those of larger diatoms [33] and increased the content of biogenic silica in the cells of smaller diatoms more than in larger diatoms [29]. However, most of these studies focused on the effects of temperature rise beyond a certain value, which prevents us from drawing a real picture of the effects of temperature rise in nature considering the seasonal variation in ambient temperature. In particular, our previous study has shown that the rise in temperature promotes the growth of larger phytoplankton assemblages more strongly at LAT and inhibits it more strongly at HAT [35]. In this study, we further found that in response to temperature rise, the smaller *diatom T. pseudonana* copes with the acute temperature rise mainly by regulating its enzyme activity and allocating a certain amount of carbon for growth, i.e., enhancing enzyme activity at LAT (Figure 5A) and conservatively regulating various metabolisms and its CUE at HAT (Figure 4E and Figure 7), while the larger *T. rotula* regulates photosynthesis and the central carbon metabolism, i.e., via up-regulation at LAT and down-regulation at HAT (Figure 4B,D and Figure 7).

### 4.1. Different Physiological Traits of Larger and Smaller Diatoms Under Different ATs

Fluctuating temperatures have a direct effect on the physiological and biochemical processes of phytoplankton, subsequently affecting their growth [16,19,49]. In winter or in high-latitude regions, lower temperatures often lead to a reduction in enzyme activities in cells, as seen in Rubisco, resulting in insufficient carbon assimilation and negatively impacting fuel metabolism and cell growth [16,50]. In contrast, in summer or in low-latitude regions, higher temperatures usually increase the half-saturation constant of Rubisco whilst promoting the production of ROS, which attack the cellular apparatus and thus reduce cell growth [51,52]. Our results support this, as shown by the higher growth rate of both the smaller diatom *T. pseudonana* and the larger *T. rotula* at MAT compared to LAT or HAT (Figure 2). In addition, our results show that more POC can be retained in cells at LAT (Figure 3E,F). This suggests that the diatoms, e.g., *Nitzschia lecointei* and *Fragilariopsis cylindrus*, are directed to synthesize and accumulate carbohydrates, polyols, amino acids and their derivatives, which serve as protective molecules to maintain their cell integrity and physiological function at LAT [53,54]. Moreover, Pg and Rd were higher in both smaller and larger cells at HAT, decoupling their lower μ values, which is consistent with our previous findings in summer [35].

Cell size is one of the most important factors influencing the response of phytoplankton to environmental changes [24]. Smaller cells usually have a higher surface area-to-volume ratio, higher efficiency of nutrient uptake and lower cost of adapting their cell structure and biochemical components to environmental changes [29,38,55]. Compared to larger cells, smaller cells at LAT had a lower pigment content along with lower photosynthetic performance, CUE and antioxidant activity (Figure 3A,C, Figure 4A,E and Figure 5A), whereas smaller cells at HAT regulated more conservatively by maintaining a higher pigment content and CUE. Previous studies have shown that maintaining a sufficient proportion of carbon for growth helps diatoms to acclimate to environmental changes [49]. Therefore, smaller cells were able to maintain growth and better acclimatize to the HAT. We further found that the pigment content, CUE, SOD activity and MDA content were higher at LAT in larger cells than in smaller cells (Figure 3B,D, Figure 4F and Figure 5B,D), suggesting that photosynthesis in larger cells supports growth more effectively at LAT, accompanied by higher oxidative pressure. The higher Chl *a* and Car contents in larger cells at LAT are inconsistent with their lower photosynthetic performance (Figure 3B,D and Figure 4B). It has been reported that some pigments can protect cells from oxidative damage; for example, Chl *a* could dissipate excess excitation energy in the form of heat or form ^3^Chl quenching through close association with Car [56], and Car could directly scavenge ROS [57]. Thus, in larger cells, Chl *a* and Cars serve to dissipate excitation energy as well as harvest light energy for the photosystems, contributing themselves to be able to cope with the higher oxidation pressure.

### 4.2. The Different Responses of Smaller and Larger Diatoms to the Temperature Rise at Different ATs

The tolerance threshold of phytoplankton to low and high temperatures determines their response to a rise in temperature at different ATs [16]. In the LAT scenario, a rise in temperature results in improvements in membrane fluidity and a reduction in the inhibition of enzyme activities, thereby promoting physiological activities, e.g., photosynthesis and respiration, and thus accelerating cell growth [15,17]. In contrast, a rise in temperature in the HAT scenario results in a decreased CO_2_ affinity of Rubisco and stronger deactivation of photosynthetic capacity compared to respiration, leading to lower carbon assimilation and thus slowing down or even stopping cell growth [21,22,50,51]. Accordingly, our results show that a rise in temperature increases the μ and metabolic rates (Pg and Rd) of both the smaller *T. pseudonana* and the larger *T. rotula* at LAT, but inhibits them at HAT (Figure 2 and Figure 4).

Cell size also affects the response of phytoplankton to the rise in temperature; for example, as the surface seawater temperature increases from year to year, smaller phytoplankton cells gradually dominate in the low and mid-latitude oceans [21,35,58,59], while larger cells dominate in high-latitude oceans [60,61]. Theoretically, smaller cells are resistant to the rise in temperature at HAT, whereas larger cells at LAT benefit more from it. Consistent with this, our results show stronger promotion of μ at LAT and stronger inhibition at HAT in larger cells compared to smaller cells (Figure 2). Our results also show that in response to the temperature rise at LAT, the input of smaller cells with a greater increase in photosynthesis was less than that of larger cells with a greater activation of respiration. The larger cells utilized almost half of their stored POC content (Figure 3F and Figure 4). In response to the temperature rise at HAT, smaller cells with only an 8% decrease in CUE and an increase in POC content were more stable than larger cells with a greater decrease in CUE and a reduction in POC content (Figure 3E,F and Figure 4E,F). This is consistent with previous findings revealing that a rise in temperature leads to the miniaturization of phytoplankton communities [24] and is a further indication of the intrinsic physiological regulation mechanisms. However, the decrease in MDA content in larger cells due to the temperature rise at HAT seems to contradict this conclusion, which could be related to the processes of ROS production and elimination. ROS are generated by electron leakage to O_2_ during electron transport in chloroplasts [62], mitochondria and plasma membranes [63], and are scavenged via two pathways, i.e., enzymatic, including SOD, ascorbate peroxidase, etc. [64], and non-enzymatic, including glutathione, Cars, peroxisome, etc. [65,66]. The decline in Pg and Rd (Figure 4B,D) and the increased SOD activity and Car content in larger cells suggest that the increased antioxidant capacity is sufficient to quench the ROS generated by the decreased electron leakage.

Transcriptome analysis also revealed the underlying metabolic mechanisms of smaller and larger cells in response to temperature rises at different ATs. In general, diatoms regulate photosynthesis and central carbon metabolism to adapt to environmental changes [49,67,68,69]. The light-harvesting complexes absorb the light energy and drive the electrons to transport through the photosystem, which produces ATP and NADPH for the Calvin cycle, enabling it to synthesize photosynthate [55]. Subsequently, the TCA cycle, glycolysis and the pentose phosphate pathway convert organic components into metabolic precursors and energy to maintain cell growth, reproduction and metabolism [49,67]. In this study, *LHCA1* and *LHCA4* of photosynthetic antenna, *PsbO*, *PsbA*, *PetH*, *PetJ*, *PasA*, *PsaF*, *AtpD* and *AtpG* of photosynthesis, *PGK*, *FBP* and *GAPDH* of carbon fixation and G6PI of Glycolysis/Gluconeogenesis were down-regulated in smaller cells at LAT (Figure 7A and Figure S1A), supporting the findings of increased metabolic rates. This difference could be due to the fact that smaller cells acclimate to the temperature rise through increased enzyme activity (Figure 5A). In contrast, these genes and their enriched KEGG pathways were up-regulated in larger cells (Figure 7B and Figure S1A), consistent with the increased metabolic rates and suggesting that larger cells are more likely to respond to the temperature rise through transcriptional regulation of functional genes (Figure 6D and Figure S1A), which also explains why larger cells are more sensitive to temperature changes due to their high metabolic cost. At HAT, the *FBP* of carbon fixation was down-regulated in smaller cells (Figure 7A and Table S1), indicating a weakening of energy conversion. Down-regulated photosynthesis usually leads to down-regulation of the TCA cycle, glycolysis and amino acid metabolism, which changes the strategy of carbon allocation [49,67]. There is a compelling notion that diatoms gain more energy by regulating the balance between different carbon metabolisms (e.g., TCA cycle, glycolysis and fatty acid degradation) to meet the challenge of temperature rise [12]. Our results support this conclusion as the key genes for carbon metabolism, such as *CS*, *SDH* and *MDH,* were changed insignificantly in the TCA cycle, while the key genes for amino acid metabolism were up-regulated in smaller cells (Figure 7A and Table S1), suggesting greater carbon availability for the biosynthesis of metabolic precursors, such as amino acids, and consequently less carbon availability for growth (Figure 2A and Figure 3E). In contrast, the genes crucial for carbon metabolism in larger cells, such as those involved in the photosynthetic antenna, photosynthesis and the TCA cycle, are extensively inhibited by higher temperatures (Figure 7B and Figure S1C), which may be one of the reasons why temperature rise drives the miniaturization of phytoplankton [13,24]. Furthermore, the up-regulation of DEGs for protein synthesis indicates that larger cells respond to the temperature rise at HAT via self-regulation, which is not just passively down-regulated.

## 5. Conclusions

In this study, we found that, compared to the smaller *T. pseudonana*, the larger *T. rotula* benefited more from the temperature rise at lower ambient temperatures by investing more in the up-regulation of their central carbon metabolism and the extensive promotion of respiration, such that almost half of the POC content was degraded and utilized for greater promotion of μ. Compared to larger cells, smaller cells were more resistant to the temperature rise at higher ambient temperatures by conservatively regulating their central carbon metabolism and balancing photosynthesis and respiration, ensuring less inhibition of μ. Our results shed light on the reason for the miniaturization of the phytoplankton community in nature, i.e., conservative regulation in smaller cells due to temperature rise at intermediate and higher ambient temperatures, and suggest that larger cells can outcompete their smaller counterparts by investing more when the temperature rise occurs at lower ambient temperature. Future studies on the effects of temperature rise on smaller and larger cells at different ATs should include experiments with different algal phyla to verify whether the strategic differences are universal across different cell sizes.

## Data Availability

The original contributions presented in this study are included in the article. Further inquiries can be directed to the corresponding authors.

## Supplementary Materials

The following supporting information can be downloaded at https://www.mdpi.com/article/10.3390/microorganisms13071652/s1, Figure S1: KEGG enrichment analysis of differentially expressed genes (DEGs) in smaller *T. pseudonana* and larger *T. rotula* responding to the increased temperature from three ambient temperatures (A–C); Table S1: The significantly enriched KEGG items in the transcriptome of *T. pseudonana* and *T. rotula*; Table S2: The temperature rise-induced differentially expressed genes (DEGs) involved in the carbon metabolic pathways of *T. pseudonana* and *T. rotula*.

## Abbreviations

The following abbreviations are used in this manuscript:

LAT	Low ambient temperature
MAT	Medium ambient temperature
HAT	High ambient temperature
ROS	Reactive oxygen species
NCMP	Provasoli-Guillard National Center of Marine Phytoplankton
CCMA	Center for Collections of Marine Algae
Chl *a*	Chlorophyll *a*
Car	Carotenoid
POC	Particulate organic carbon content
MDA	Malondialdehyde
SOD	Superoxide dismutase
Pg	Gross photosynthetic O_2_ evolution rate
Rd	Dark respiration rate
CUE	Carbon use efficiency
DEGs	Differentially expressed genes
Nr	NCBI non-redundant protein sequences
Swiss-Prot	Manually annotated and reviewed protein sequence database
KEGG	Kyoto Encyclopedia of Genes and Genomes database
FC	Foldchange
*PsbO*	Manganese-stabilizing protein
*PsbA*	Photosystem II reaction center protein D1
*PsbM*	Photosystem II reaction center protein M
*PetH*	Ferredoxin--NADP reductase
*PetJ*	Cytochrome c6
*LHCA1*	Photosystem I chlorophyll a/b-binding protein 1
*LHCA4*	Photosystem I chlorophyll a/b-binding protein 4
*PsaA*	Photosystem I P700 chlorophyll a apoprotein A1
*PsaF*	Photosystem I reaction center subunit III
*AtpD*	ATP synthase subunit delta
*AtpG*	ATP synthase gamma chain
*PGK*	Phosphoglycerate kinase
*GAPDH*	Glyceraldehyde-3-phosphate dehydrogenase
*FBP*	Fructose-1,6-bisphosphatase
*PRK*	Phosphoribulokinase
*Rubisco*	Ribulose bisphosphate carboxylase
*G6PI*	Glucose-6-phosphate 1-epimerase
*PK*	Pyruvate kinase
*ripA*	Ribose-5-phosphate isomerase 1
*PRPS*	Ribose-phosphate pyrophosphokinase 1
*PC*	Pyruvate carboxylase
*CS*	Citrate synthase
*OGDH*	2-oxoglutarate dehydrogenase E1 component
*LSC*	Succinyl-CoA synthetase alpha subunit
*SDH*	Succinate dehydrogenase
*MDH*	Malate dehydrogenase
*ND*	2-oxoglutarate dehydrogenase E1 component
*ATP5F1A*	ATP synthase subunit alpha
*ATP5F1C*	ATP synthase subunit gamma
*Prx*	Peroxiredoxin
*VDE*	Violaxanthin de-epoxidase
*ZEP*	Zeaxanthin epoxidase
*FCP*	Fucoxanthin-chlorophyll protein
