# Supplementary material for: Responses of Different Temperature-Acclimated Diatom Species, Smaller Thalassiosira pseudonana and Larger Thalassiosira rotula, to Increased Ambient Temperature
Source: Microorganisms. 2025 Jul 12;13(7):1652. doi: 10.3390/microorganisms13071652 (PMC12298872; doi:10.3390/microorganisms13071652)
Supplement: Supplementary file 1 [file microorganisms-13-01652-s001.zip › microorganisms-3678767-supplementary.pdf]

Supplementary File

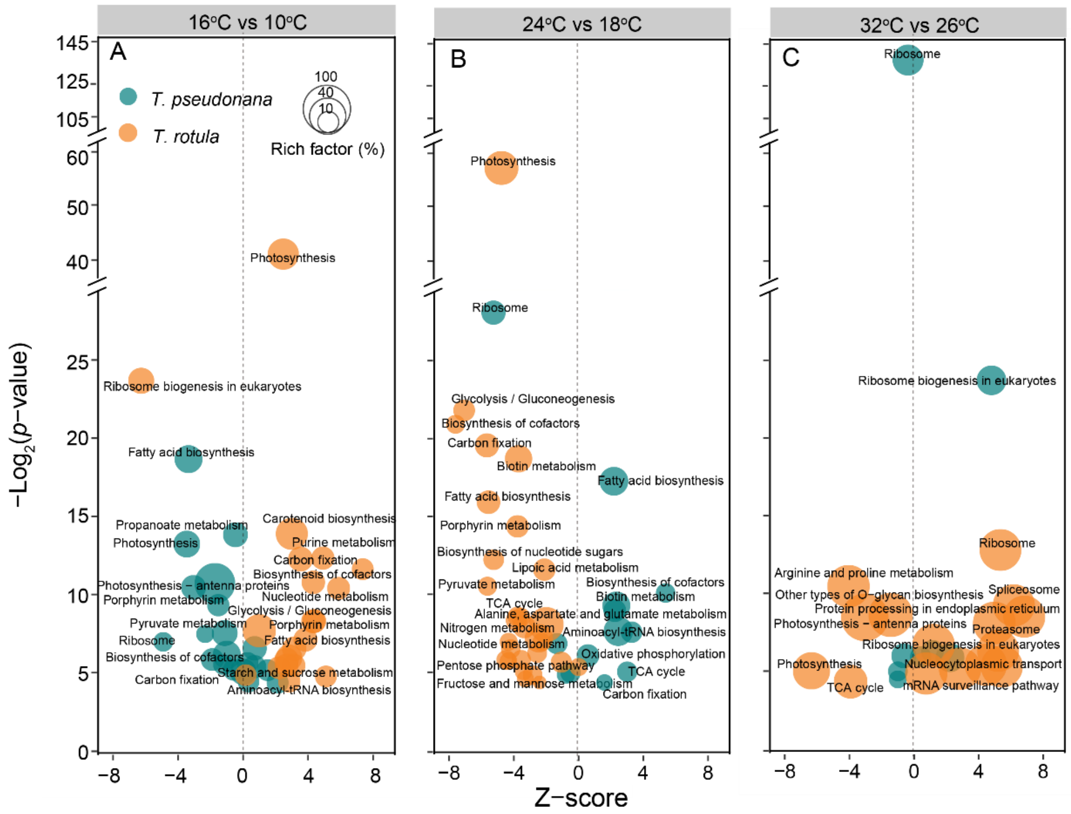

Figure S1. KEGG enrichment analysis of differentially expressed genes (DEGs) in smaller *T. pseudonana* and larger *T. rotula* responding to the increased temperature from three ambient temperatures (A-C).

Table S1 The significantly enriched KEGG items in the transcriptome of *T. pseudonana* and *T. rotula*.

T. pseudonana				*T. rotula*			
ID	Pathway	*p* value	Z score	ID	Pathway	*p* value	Z score
16 ºC vs 10 ºC				16 ºC vs 10 ºC			
ko00061	Fatty acid biosynthesis	<0.001	-3.36	ko00195	Photosynthesis	<0.001	2.47
ko00640	Propanoate metabolism	<0.001	-0.47	ko03008	Ribosome biogenesis in eukaryotes	<0.001	-6.27
ko00195	Photosynthesis	<0.001	-3.46	ko00906	Carotenoid biosynthesis	<0.001	3
ko00196	Photosynthesis - antenna proteins	<0.001	-1.73	ko00230	Purine metabolism	<0.001	4.91
ko00860	Porphyrin metabolism	<0.001	-3.05	ko00710	Carbon fixation	<0.001	3.54
ko00620	Pyruvate metabolism	0.0016	-1.53	ko01240	Biosynthesis of cofactors	<0.001	7.36
ko00730	Thiamine metabolism	0.0054	-1.13	ko01232	Nucleotide metabolism	<0.001	4.32
ko01240	Biosynthesis of cofactors	0.0056	-2.33	ko00010	Glycolysis / Gluconeogenesis	<0.001	5.88
ko03010	Ribosome	0.0079	-4.91	ko00860	Porphyrin metabolism	0.003	4.38
ko00760	Nicotinate and nicotinamide metabolism	0.0106	0.71	ko00061	Fatty acid biosynthesis	0.003	4.31
ko01040	Biosynthesis of unsaturated fatty acids	0.014	-1.00	ko00196	Photosynthesis - antenna proteins	0.005	1
ko00710	Carbon fixation	0.017	-1.90	ko00400	Phenylalanine, tyrosine and tryptophan biosynthesis	0.007	3.84
ko01232	Nucleotide metabolism	0.020	0.28	ko00450	Selenocompound metabolism	0.011	3.15
ko00010	Glycolysis / Gluconeogenesis	0.022	-1.21	ko00960	Tropane, piperidine and pyridine alkaloid biosynthesis	0.016	2.83
ko00564	Glycerophospholipid metabolism	0.024	0.71	ko00730	Thiamine metabolism	0.018	2.67
ko00260	Glycine, serine and threonine metabolism	0.028	-0.58	ko00900	Terpenoid backbone biosynthesis	0.023	2.68
ko00280	Valine, leucine and isoleucine degradation	0.028	1.51	ko00500	Starch and sucrose metabolism	0.023	3.13
ko00740	Riboflavin metabolism	0.030	0	ko00030	Pentose phosphate pathway	0.025	2.2
ko00965	Betalain biosynthesis	0.039	-1.00	ko03018	RNA degradation	0.035	0.16
ko00908	Zeatin biosynthesis	0.040	-1.00	ko00970	Aminoacyl-tRNA biosynthesis	0.037	5.10
24 ºC vs 18 ºC				24 ºC vs 18 ºC			

ko03010	Ribosome	<0.001	-5.26	ko00195	Photosynthesis	<0.001	-4.77
ko00061	Fatty acid biosynthesis	<0.001	2.18	ko00010	Glycolysis / Gluconeogenesis	<0.001	-7.07
ko01240	Biosynthesis of cofactors	<0.001	5.37	ko01240	Biosynthesis of cofactors	<0.001	-7.60
ko00780	Biotin metabolism	0.0016	2.33	ko00710	Carbon fixation	<0.001	-5.67
ko00250	Alanine, aspartate and glutamate metabolism	0.0020	2.24	ko00780	Biotin metabolism	<0.001	-3.71
ko00970	Aminoacyl-tRNA biosynthesis	0.0053	3.27	ko00061	Fatty acid biosynthesis	<0.001	-5.57
ko00740	Riboflavin metabolism	0.0051	2.45	ko00860	Porphyrin metabolism	<0.001	-3.77
ko00520	Amino sugar and nucleotide sugar metabolism	0.0087	-1.34	ko01250	Biosynthesis of nucleotide sugars	<0.001	-5.25
ko00910	Nitrogen metabolism	0.0149	0.58	ko00785	Lipoic acid metabolism	<0.001	-2.12
ko01250	Biosynthesis of nucleotide sugars	0.032	-0.50	ko00620	Pyruvate metabolism	<0.001	-5.63
ko00190	Oxidative phosphorylation	0.035	-0.78	ko00250	Alanine, aspartate and glutamate metabolism	0.003	-3.78
ko00020	TCA cycle	0.031	3.00	ko00020	TCA cycle	0.003	-3.57
ko00710	Carbon fixation	0.042	1.50	ko00400	Phenylalanine, tyrosine and tryptophan biosynthesis	0.003	-3.84
				ko00196	Photosynthesis - antenna proteins	0.004	-2.00
				ko00730	Thiamine metabolism	0.004	-3.36
				ko00906	Carotenoid biosynthesis	0.007	-2.45
				ko00520	Amino sugar and nucleotide sugar metabolism	0.008	-4.32
				ko00630	Glyoxylate and dicarboxylate metabolism	0.008	-2.29
				ko00030	Pentose phosphate pathway	0.011	-4.20
				ko00910	Nitrogen metabolism	0.013	-2.56
32 ºC vs 26 ºC				32 ºC vs 26 ºC			
ko03010	Ribosome	<0.001	-0.37	ko03010	Ribosome	<0.001	5.35
ko03008	Ribosome biogenesis in eukaryotes	<0.001	4.80	ko00330	Arginine and proline metabolism	<0.001	-4.05
ko00261	Monobactam biosynthesis	0.0068	0	ko03040	Spliceosome	0.0017	6.18
ko00260	Glycine, serine and threonine metabolism	0.013	1.15	ko00514	Other types of O-glycan biosynthesis	0.003	-1.44
ko00920	Sulfur metabolism	0.016	-0.63	ko04141	Protein processing in	0.003	6.83

					endoplasmic reticulum		
ko00630	Glyoxylate and dicarboxylate metabolism	0.017	0.41	ko00196	Photosynthesis - antenna proteins	0.003	-3.05
ko00750	Vitamin B6 metabolism	0.017	2.24	ko03050	Proteasome	0.004	4.97
ko00010	Glycolysis / Gluconeogenesis	0.032	-1.00	ko03008	Ribosome biogenesis in eukaryotes	0.013	5.21
ko00620	Pyruvate metabolism	0.045	-1.00	ko03015	mRNA surveillance pathway	0.024	4.45
ko00710	Carbon fixation	0.048	-1.34	ko03013	Nucleocytoplasmic transport	0.026	5.44
				ko03082	ATP-dependent chromatin remodeling	0.028	2.85
				ko00195	Photosynthesis	0.046	-7.04
				ko04146	Peroxisome	0.047	0.89
				ko00020	TCA cycle	0.049	-4.75

Table S2 The temperature rise-induced differentially expressed genes (DEGs) involved in the carbon metabolic pathways of *T. pseudonana* and *T. rotula*.

Pathway	Genes name	*T. pseudonana* log$_2$FC (LAT)	log$_2$FC (MAT)	log$_2$FC (HAT)	Genes name	*T. rotula* log$_2$FC (LAT)	log$_2$FC (MAT)	log$_2$FC (HAT)
Photosynthesis	*PsbO*	-1.77*	0.82	-0.23	*PsbO*	1.92*	-1.19*	13.53*
	PsbA	-1.07*	-0.09	0.45	*PsbM*	2.23*	-2.21*	-13.61*
	PetH	-1.50*	0.21	-0.53	*PetH*	0.98	-0.79	-15.27*
	PetJ	-1.77*	0.51	-0.53	*PetJ*	2.40*	-0.78	-14.97*
	PsaA	-2.24*	-0.70	-0.53	*LHCA1*	2.36*	-2.00*	-12.82*
	PsaF	-2.02	-0.89	1.22	*LHCA4*	2.08*	-2.30*	14.97*
	AtpD	-1.40*	-0.56	-0.37	*AtpA*	1.00*	-2.13*	-2.80*
	AtpG	-1.24*	0.41	-0.23	*AtpG*	0.84	-0.38	-14.04*
Calvin Cycle	*PGK*	-3.37*	0.86	-0.40	*PGK*	1.54*	-0.66	-13.52*
	GAPDH	-1.45*	0.87	0.27	*GAPDH*	1.22*	-1.15*	-3.76*
	FBP	-3.77*	2.12*	-1.38*	*FBP*	0.52	-2.38*	-6.43*
	PRK	-2.53	1.61*	-2.75	*PRK*	4.46*	-1.65*	-15.05*
	Rubisco	-0.64	-1.11	-1.01	*Rubisco*	-0.69	-0.97	-1.08
Glycolysis/Gluconeogenesis	*G6PI*	-1.23*	0.316	-0.17	*G6PI*	1.82*	-1.72*	-9.90*
	PK	0.29	-0.32	-0.56	*PK*	0.68	-0.95	-15.64*
Pentose phosphate pathway (PPP)	*ripA*	-0.03	0.25	-0.64	*ripA*	1.32*	-3.09*	-6.23*
	PRPS	-0.11	-0.55	0.06	*PRPS*	1.71	-2.96*	-3.22*
TCA cycle	*PC*	-0.54	1.04*	-0.83	*PC*	1.87*	-1.29*	-14.80*
	CS	-0.69	0.35	0.26	*CS*	2.97	-4.91	-5.29*
	OGDH	1.36	1.24*	-1.45*	*OGDH*	0.92	-2.21*	-1.43*
	LSC	0.97	0.28	0.03	*DLD*	2.26*	-1.24*	-11.32*
	SDH	0.54	0.40	-0.66	*LSC*	-0.73	-0.06	-8.08*
	MDH	0.04	0.56	0.20	*MDH*	0.72	0.51	-7.85*
Oxidative phosphorylation	*ND*	-1.07*	-0.75	-0.02	*CYC*	0.57	-0.17	-16.72*
	ATP5F1A	-0.25	-0.32	0.01	*ATP5F1A*	1.02*	-0.31	-15.72*
	ATP5F1C	-0.16	-0.78	-0.01	*ATP5F1E*	0.55	0.02	-14.22*
Peroxisome	*SOD*	-0.40	0.05	-0.39	*SOD*	0.11	0.08	3.30*
	Prx	0.22	-0.17	-0.63	*Prx*	0.27	1.75	3.14*
Non-photochemical quenching (NPQ)	*VDE*	-0.87	0.62	-0.43	*FCP*	-2.10*	-1.22*	-8.24*

ZEP	-1.41*	-0.51	0.06
